# Supplementary figures and images for: A protease protection assay for the detection of internalized alpha-synuclein pre-formed fibrils
Source: PLoS One. 2021 Jan 26;16(1):e0241161. doi: 10.1371/journal.pone.0241161 (PMC7837481; doi:10.1371/journal.pone.0241161)

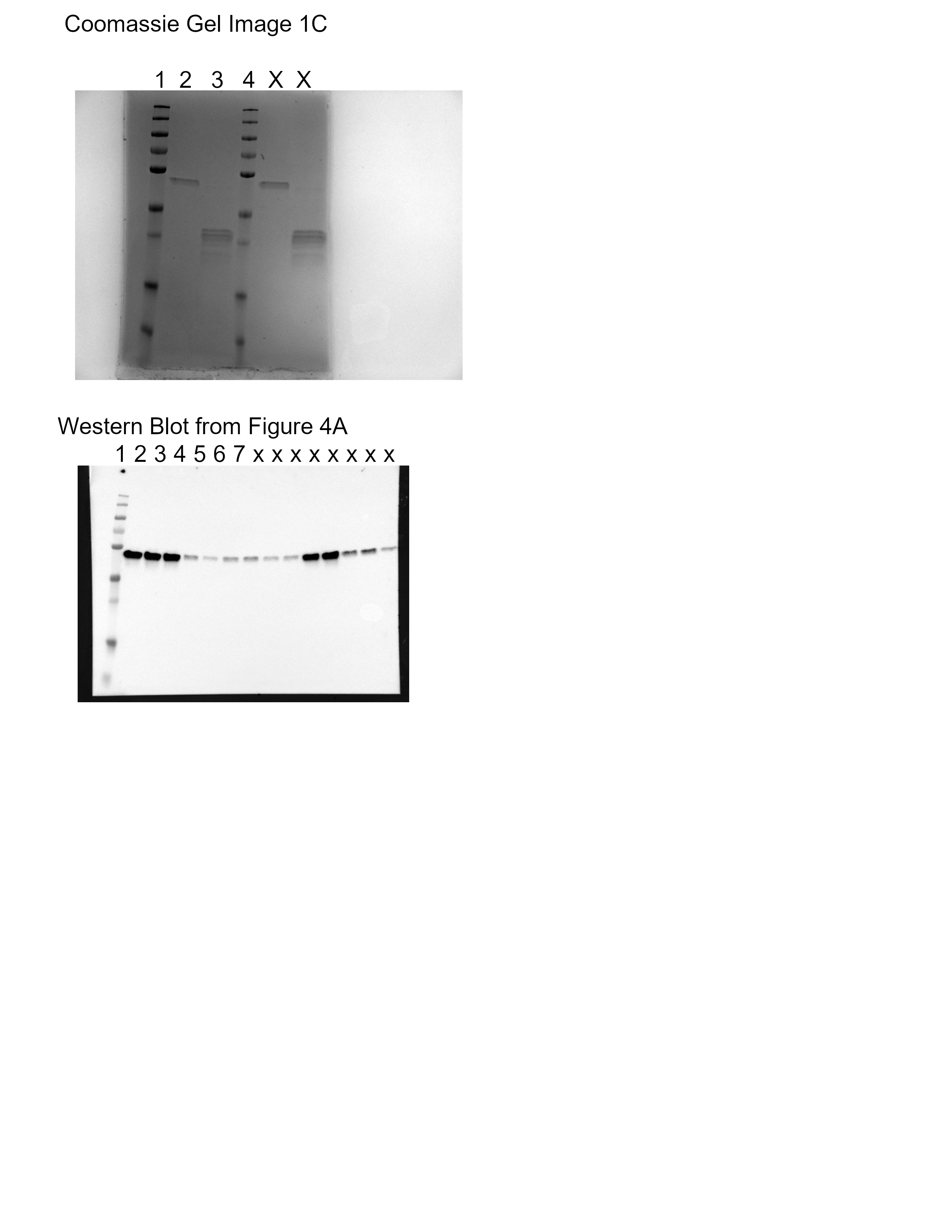

Supplement: S1 Raw images — (TIF) [file pone.0241161.s001.tif]
