# Supplementary material for: A protease protection assay for the detection of internalized alpha-synuclein pre-formed fibrils
Source: PLoS One. 2021 Jan 26;16(1):e0241161. doi: 10.1371/journal.pone.0241161 (PMC7837481; doi:10.1371/journal.pone.0241161)
Supplement: Attachment — Submitted filename: rebuttalsubmitted (2).docx [file pone.0241161.s002.docx]

**Response to Reviewers:**

We thank the reviewers for the time spent on our manuscript and have revised the paper to specifically address all of their comments, as detailed below, including performing the two new experiments requested by Reviewer 2. We hope that the revised paper is now acceptable for publication.

**Reviewer #1:** This is an interesting and important study describing a useful methodology for generation of the α-synuclein pre-formed fibrils (PFFs) suitable for the analysis of the cellular processes underlying cell-to-cell transmission of α-synuclein proteopathic aggregates. This is achieved by the incorporation of a tobacco etch virus (TEV) protease cleavage site between α-synuclein and green fluorescent protein. Utilization of this construct allows for the efficient removal of GFP from the non-internalized PFFs. Although the presented data are convincing, the authors should add a short discussion of the fact that this approach does not exclude a possibility for the α-synuclein PFFs with the removed GFPs to interact with the cell, be internalized, and affect cellular processes in parallel with the internalized GFP-labeled PFFs.

We have now expanded our mention of this subject in the Discussion as follows (and we thank the reviewer for his/her positive remarks).

A key limitation of our method is the fact that TEV treatment removes the fluorescent label but leaves α−synuclein still attached to the cell surface; thus, α-synuclein species lacking GFP may still continuously enter the cell while the internalized fluorescent species is under examination. **This internalized α-synuclein may affect cellular processes in parallel with the internalized GFP-labeled PFFs**. This is also a limitation of the trypan blue technique: while outside fluorescence is quenched, GFP-PFFs still remain on the cell surface and are presumably continuously internalized.

**Reviewer #2:** In the present manuscript, Jarvela et al provided a new tool to detect the endocytosed pre-formed alpha-synuclein fibrils. Given the importance of cell-to-cell transmission of alpha-synuclein Pff in Parkinson’s disease pathophysiology, it is interesting to develop the method visualizing transmitted Pff at the cellular level for further mechanism research. However, it is still many critical flaws to reach the author’s conclusion, in particular, missing many control experiment. Also, considering the relatively short text length, there are many mislabeling everywhere.

*Major points:*

1. The authors used alpha-synuclein construct inserted with His, TEV-p site, and GFP, and then tested pre- and post-TEV treatment effect in most experiments. Since TEV-p site is critical for this new tool development, it should be included No-TEV-p site construct as a negative control.

This experiment now includes the requested no-TEV control (new **Figure 6B**).

2. In Fig3, the authors conclude that TEV-P treatment removes extracellular GFP signal as effectively as trypan blue quenching. However, despite the statistical insignificance in Fig 3-B, it looks like a huge difference between pre- and post-quenching after TEV treatment (Fig 3-A bottom image). It would be necessary to show that the GFP-positive signals in the pre-quench (+TEV) is not a membrane-localized signal using a membrane marker antibody.

We are confused by this last statement, as we DO believe that the GFP-positive signal in the pre-quench sample is membrane-localized. The affinity of the sticky protein synuclein for membranes has also amply been demonstrated by others. Regarding the post-quench GFP signal, the majority of this fluorescence is clearly intracellular (see Figure 3A, Figure 6).

3. In Fig 4, the authors claim that exposure of cells to TEV-P removes 95% of alpha-syn-TEV-GFP signals using western blot. But it may possible that TEV-P protein can enter the cells and induce proteolysis of the endocytosed proteins. Therefore, it needs to prove that TEV-P treatment is solely working on the extracellular absorbed proteins using a negative control expression. As the authors discussed, if this method can be useful for the endocytosed Pff assay tool, the TEV-Protease should not enter cells freely.

We simply do not have a method to answer this point, so we have added a caveat sentence to the Results to mention the possibility that the GFP cleavage reaction might occur inside the cells rather than outside (line 298). However, we feel this possibility is quite remote given the fact that cells are exposed to dilute protease for only 30 min. Intracellular TEV proteolysis would imply a rapid and robust uptake of enzyme followed by substantive internal encounter with PFF substrate in the crowded intracellular environment**.** This reaction is unlikely to be quantitatively important compared to the readily available surface-associated cleavage reaction.

4. In Fig6-A, there is no quantification data.

We have now performed this quantification (new **Figure 6A, right portion**).

5. In Fig6-B, considering long exposure of pff on the cultured neurons, authors should include the TEV-P no treatment set as a negative control with quantification data.

This experiment has now been performed with the requested control (new **Figure 6C**). Unfortunately, only tiny quantities of internalized PFFs remain after 4 days, thus making reliable quantification impossible. Interestingly, in this long-term experimental paradigm, TEV treatment 4 days prior to fixation apparently does not reduce intracellular PFF fluorescence. We speculate that in the absence of TEV, many more GFP-PFFs remain surface-attached, and these are then persistently endocytosed over the 4-day incubation period. In contrast, cells exposed to PFFs incubated with TEV undergo rapid cleavage of the fluorescent fusion protein; thus little to no fluorescent PFFs remain to be internalized over time. These data support the idea that the TEV technique is most useful over short time frames. The text has now been altered to reflect this idea.

*Minor points:*
-In many places, authors used a-syn or a-synuclein instead of α-syn or α-synuclein. We were unable to find any instances of use of **a**-syn or **a**-synuclein in the ms except in the abstract; we have now substituted “alpha” since we cannot use symbols in the abstract..
-In many places, the authors used both “ⅹ” and “X” (e.g. 40X oil). Need to make it consistent. Changed
-In Fig 5, there is no statistical marks. changed
-line 66, need to check “ENLYFQˇG” changed to residues only
-line 74, 50ug/ml change to 50μg/ml changed
-line 110, 37° C change to 37°C changed
-line 110, CO2 change to CO2 (lower case) changed
-line 135, 4 C change to 4°C changed
-line 141, 35-mM change to 35-mm changed
-line 159, 164, 260, 37 C change to 37°C changed
-line 276, incorrect labeling of (top) and (bottom) corrected
